# Over-expression of microRNA-145 drives alterations in β-adrenergic signaling and attenuates cardiac remodeling in heart failure post myocardial infarction

**DOI:** 10.18632/aging.103320

**Published:** 2020-06-18

**Authors:** Zhebo Liu, Bo Tao, Suzhen Fan, Shengyu Cui, Yong Pu, Liqiang Qiu, Hao Xia, Lin Xu

**Affiliations:** 1 Department of Cardiology, Renmin Hospital of Wuhan University, Wuhan, PR China; 2 Cardiovascular Research Institute, Wuhan University, Wuhan, PR China; 3 Hubei Key Laboratory of Cardiology, Wuhan, PR China; 4 Renmin Hospital of Hannan, Renmin Hospital of Wuhan University, Wuhan, PR China

**Keywords:** microRNA-145, β-adrenergic signaling, cardiac remodeling, heart failure, myocardial infarction

## Abstract

Background: Numerous studies have highlighted the crucial role of microRNA-145 (miR-145) in coronary atherosclerosis and myocardial ischemia reperfusion injury. However, effects of miR-145 on β-adrenergic signaling and cardiac remodeling in heart failure (HF) remains unclarified.

Methods and Results: We established HF model in rats with left anterior descending coronary artery (LAD) occlusion. Four weeks after LAD ligation, rats showed substantial aggravation of cardiac dilation and electrophysiological instability. Up-regulation of miR-145 ameliorated HF-induced myocardial fibrosis and prolonged action potential duration. Echocardiography revealed increased basal contractility and decreased left ventricular inner-diameter in miR-145 over-expressed heart, while cardiac response to β-adrenergic receptor (βAR) stimulation was reduced. Furthermore, miR-145 increased L-type calcium current (I_Ca_) density while decreased I_Ca_ response to β-adrenergic stimulation with isoproterenol. The alterations in βAR signaling might be predominant due to miR-145-mediated activation of Akt/CREB cascades. At high frequency pacing, Ca^2+^ transient, cell shortening and frequency of Ca^2+^ waves were significantly improved in AD-miR-145 group. Western blotting revealed that increased expression of Ca_v_1.2, Ca^2+^-ATPase, β2AR, GNAI3 and decreased level of CaMKII might be attributed to the cardioprotective effects of miR-145.

Conclusion: miR-145 effectively alleviates HF-related cardiac remodeling by improving cardiac dilation, fibrosis, intracellular Ca^2+^ mishandling and electrophysiological instability.

## INTRODUCTION

Cardiac remodeling, which consists of electrical remodeling, structural remodeling and neural remodeling underlies the pathophysiological basis of heart failure (HF) [1, 2]. Despite great advances in drug and device therapy, excessive and irreversible remodeling remain a major threat to the prognosis of HF patients [3]. Angiotensin converting enzyme inhibitors (AECI) and β-adrenergic receptor (βAR) antagonist can delay the progress of adverse remodeling. However, their clinical applications are limited in some patients due to adverse effects, such as hyperkalemia and hypotension. Therefore, more safe and effective therapeutic targets are sought imperatively for the prevention of cardiac remodeling.

MicroRNAs (miRs) are highly conserved endogenous non-coding RNAs of around 23 nucleotides in length, and negatively modulate the expression of messenger RNA (mRNA) by suppressing translation or cleavage of multiple target mRNAs [4, 5]. Among miRs, miR-145 is abundantly expressed in cardiovascular system with various functions. Evidence showed that miR-145 could protect cardiomyocytes against apoptosis induced by hydrogen peroxide [6]. In addition, we previously uncovered the cardioprotective role of miR-145 against ischemia/reperfusion injury [7]. However, in-depth mechanism regarding anti-malignant arrhythmia effects of miR-145 remains elusive. although miR-145 may represent a novel potential pharmacological therapeutic target, the underlying cardioprotective mechanism remains unclarified.

HF is featured by diminished response to catecholamines due to alterations in the βAR signaling [8]. HF-facilitated β2AR-receptor coupled GTP-binding protein i (G_i_) signaling, thereby attenuating β1AR-evoked maladaptive remodeling and cardiomyocytes apoptosis [9]. In the meantime, β1AR-mediated increases in L-type Ca^2+^ current (I_Ca_) and myocardial contractility via receptor coupled GTP-binding protein s (G_s_)-protein kinase A (PKA) cascades may be inhibited as well [9]. Ca^2+^/Calmodulin-dependent protein kinase II (CaMKII) is another key protein in the regulation of I_Ca_ and intracellular Ca^2+^ homeostasis after HF. Pathological CaMKII overactivation results in a remarkable reduction of sarcoendoplasmic reticulum Ca2+ ATPase (SERCA) expression and hyperphosphorylation of ryanodine receptor 2 (RyR2). These changes may dramatically aggravate Ca^2+^ leakage, contractile dysfunctions and cardiac hypertrophy [10, 3]. CaMKII inhibition has been proved as a potential therapy against cardiac remodeling and arrhythmia [11], and recent researches demonstrated that CaMKII was a downstream target of miR-145 [7, 12].

Therefore, we presented the following questions based on the above-mentioned observations: 1) miR-145 may acts as a crucial regulator in CaMKII signaling pathway for determining cardiac remodeling after HF; 2) whether miR-145-induced depression on CaMKII might compensatorily promote βAR-G_s_-PKA cascades, since both CaMKII and PKA activities are both required for the modulation of I_Ca_ channels and intracellular contractile myofilaments. Accordingly, we established HF models in rats with over-expressed miR-145 to verify this hypothesis. Meanwhile, the anti-remodeling validity of miR-145 was evaluated and compared with the aid of traditional medicine (combination of ACEI and βAR antagonist).

## RESULTS

### Relative expression of miR-145

**Figure 1:**
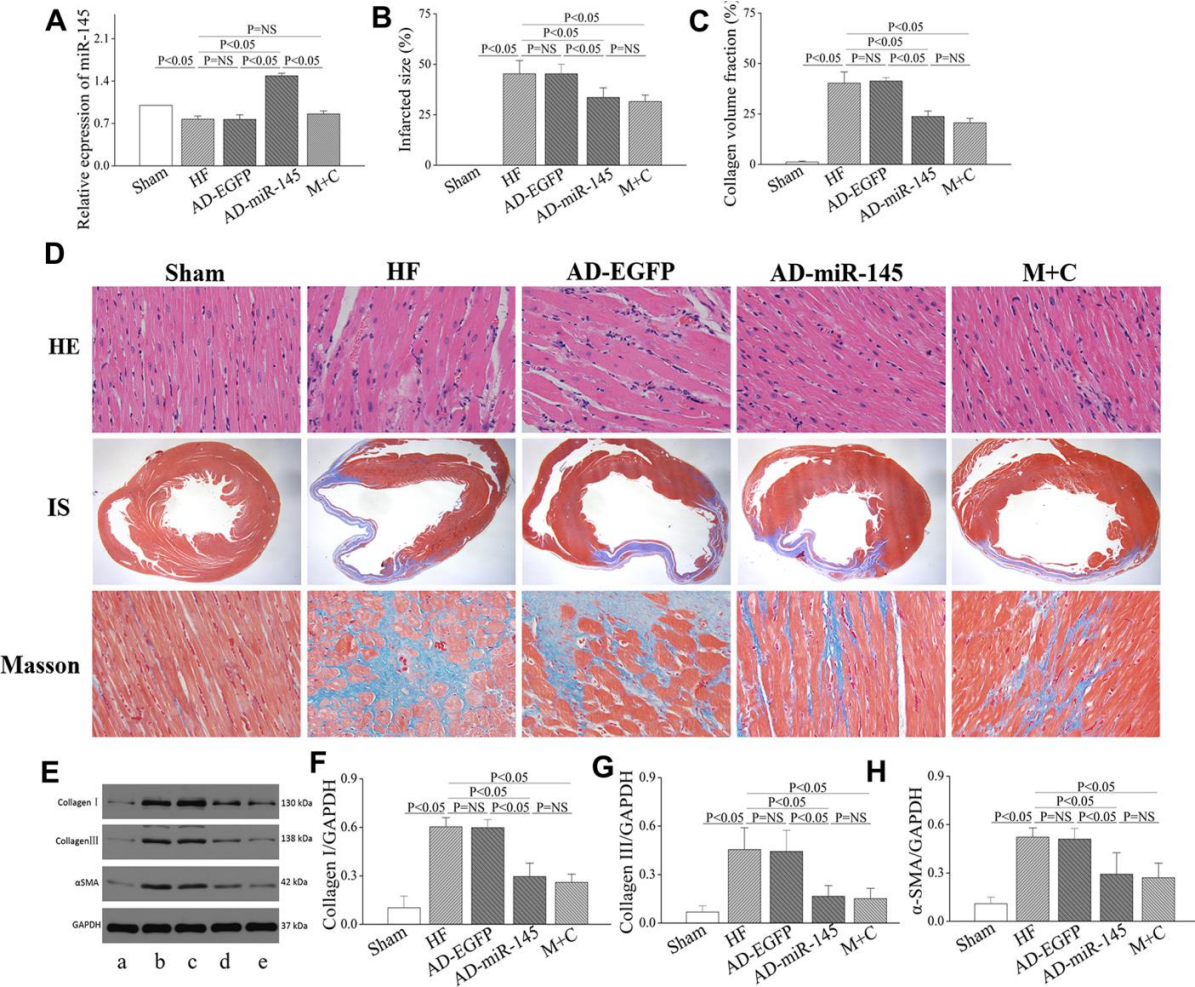
**miR-145 attenuated structural remodeling after HF.** (**A**) relative expression of miR-145 (n=3); (**B**) quantification of infarcted size (n=5); (**C**) statistical results of LV fibrosis (n=5); (**D**) representative imagine of HE and masson’s stain; (**E**) representative western blots; (**F**–**H**) quantitative analysis of the immunoreactive band displayed by bar graph (n=3). (a) sham group; (**B**) HF group; (**C**) AD-EGFP; (**D**) AD-miR-145 group; (**E**) M+C group; IS, infarcted size. Data are presented as mean ± SD.

Quantitative reverse transcription polymerase chain reaction (RT-qPCR) was used to detect the expression of miR-145 4 weeks after LAD ligation. As shown in Figure 1A, the level of miR-145 was significantly reduced in HF and AD-EGFP groups, while that was notably increased in AD-miR-145 group. These results suggested that successful transfection of miR-145 and HF could lead to a reduction in miR-145 levels.

### Structural remodeling analysis

As shown in Figure 1D, myocardial structure in HF and AD-EGFP groups exhibited disordered arrangement with cardiomyocytes hypertrophy compared with Sham group 4 weeks after HF, while these disorganizations were partly attenuated in groups treated with miR-145 and M+C. Moreover, a notably LV fibrosis was also observed after HF, as revealed by enhanced collagen volume fraction (CVF) and IS (Figure 1B–1D). In addition, the protein expressions of type I college, type III college and α-smooth muscle actin (α-SMA), as indicators of collagen deposition, were markedly increased in HF and AD- EGFP groups (Figure 1E–1H). However, up-regulation of miR-145 or M+C treatment strikingly reversed HF-induced maladaptive collagen deposition featured by diminished CVF and IS. The above-mentioned results indicated that miR-145 could effectively suppress the maladaptive structural remodeling after HF, and its effectiveness was not inferior to the combination of ACEIs and β-AR antagonis.

### Ponderal data

Weight gain of lung and heart are characteristics of advanced HF [13–15]. As shown in Table 1, HF-induced augments in lung weight (LW) and heart weight (HW) were reflected by the increase in LW/BW and HW/BW ratio respectively. However, these indicative parameters were notably alleviated in miR-145 and M+C groups. These findings highlighted that up-regulation of miR-145 was capable of attenuating HF-induced maladaptive remodeling.

**Table 1:** Ponderal data for each group (n=6).

	**Sham**	**HF**	**AD-EGFP**	**AD-miR-145**	**M+C**
BW (g)	310.45±7.06	301.93±8.91	298.7±8.89	295.51±7.28	297.24±6.76
HW/BW (mg/g)	0.32±0.01	0.40±0.02^*^	0.42±0.02^*^	0.36±0.01^*#&^	0.37±0.01^*#^
LW/BW (mg/g)	0.38±0.01	0.48±0.02^*^	0.48±0.02^*^	0.43±0.02^*#&^	0.43±0.02^*#^
HR (bpm)	442.40±15.69	426.83±7.68	421.53±11.10	432.20±14.65	407.03±16.77

BW: body weight; LW: lung weight; HW: heart weight; *P<0.05, compared with sham group; #P<0.05, compared with HF group; &P<0.05, compared with AD-EGFP group. Data are presented as mean ± SD.

### Evaluation of electrocardiogram (ECG)-based parameters

**Figure 2:**
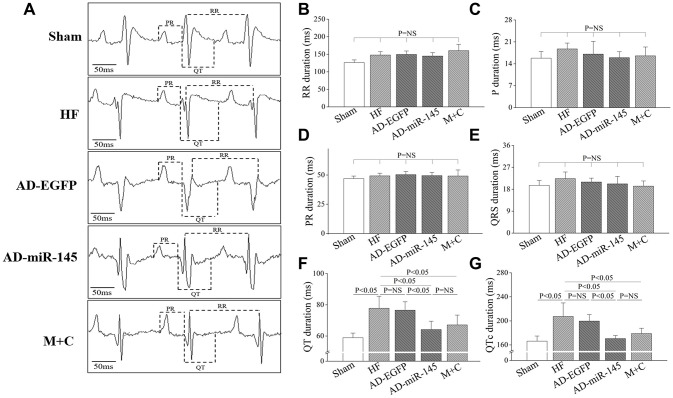
**Characters of ECG parameters.** (**A**), typical ECG recording after HF; (**B**–**G**), statistical results of P duration, RR interval, PR interval, QRS interval, QT interval and QTc interval (n=6). Data are presented as mean ± SD.

ECG lead II was recorded and analyzed. The ECG-based parameters, including P waves duration, PR interval, RR interval and QRS interval, were similar among the 5 groups (Figure 2A–2E). However, repolarization (manifested by QT and QTc interval) was prolonged in HF and AD-EGFP groups (Figure 2F and 2G), while rats in miR-145 and M+C groups showed significantly shorter in QT and QTc intervals compared with those in HF and AD-EGFP groups. These results indicated that over-expression of miR-145 could reverse HF-induced prolongation of QTc.

### Analysis of electrical remodeling

**Figure 3:**
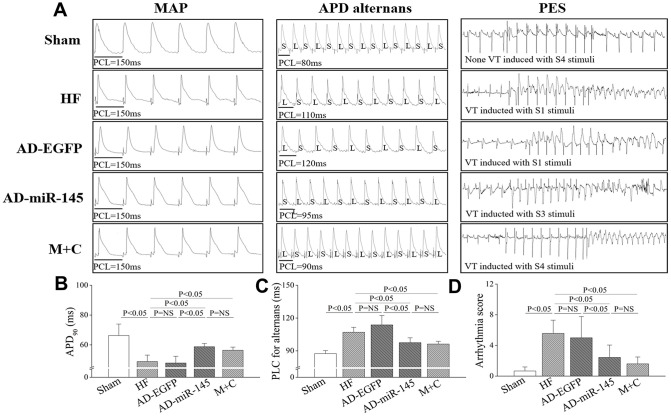
**MiR-145 depressed the electrophysiological susceptibility of HF.** (**A**) representative MAP recording at a PCL=150ms, APD alternans and PES; (**B**–**D**) statistical results of APD_90_, APD alternans threshold and arrhythmia score (n≥5); PCL, pacing cycle length; L, longer APD; S, shorter APD. Data are presented as mean ± SD.

Action potential duration (APD) dispersion along with diminished threshold of APD alternans threshold are major factors leading to reentry ventricular arrhythmia [16–17]. As shown in Figure 3A–3C, LAD ligation resulted in a noticeably shorter APD_90_ and decreased of threshold of APD alternans in HF and AD-EGFP groups. However, APD_90_ in AD-miR-145 and M+C groups was markedly prolonged along with increased threshold of APD alternans. These data suggested that miR-145 over-expression could reduce HF-induced susceptibility to malignant ventricular arrhythmia. The results were further corroborated by programmed electrical stimulation (PES). As illustrated in Figure 3A and 3D, rats in HF and AD-EGFP groups were more vulnerable to ventricular tachycardia (VT) with potentially higher arrhythmia scores, while the electrophysiological instability was ameliorated by up-regulation of miR-145 and M+C treatment. Previous study by Wang, et al. pointed out that APD_90_ might be different at none infarcted zone (NIZ) and IBZ after MI, they demonstrated that the APD_90_ in IBZ was significantly shortened while APD_90_ in NIZ was markedly prolonged after MI [17], these serious changes might eventually manifest as the prolongation of QT interval. In addition, a study by Gui, et al. also supported our results [16]. They demonstrated that APD_90_ in IBZ was significantly shortened after MI while QT interval detected by ECG was prolonged.

### Altered cardiac function in vivo and attenuated response to β-adrenergic stimulation

**Figure 4:**
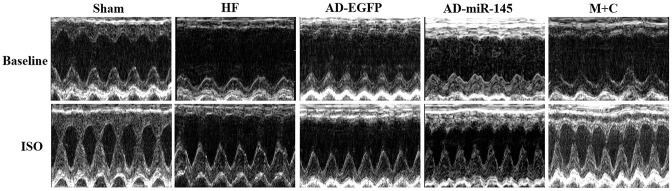
**Representative images of M-mode echocardiography at baseline and 5min after ISO injection, respectively.**

To investigate the effects of miR-145 on cardiac functions in vivo, echocardiograph was performed on rats after lightly anesthesia. Rats in HF and AD-EGFP groups showed a notable deterioration of left ventricular (LV) dilatation and contractile dysfunction, manifested by enlarged in left ventricular internal dimension (LVID), both end-systolic and end-diastolic (LVIDs and LVIDd) as well as decreasing in ejection fraction (EF) and fractional shortening (FS) (Figure 4 and Table 2). Strikingly, the above mentioned parameters of cardiac function were improved in the HF group that received miR-145 or M+C treatment. It was therefore unveiled that up-regulation of miR-145 caused protective effects against cardiac remodeling. Subsequently, we tested the cardiac response to β- adrenergic stimulation via intraperitoneal injection of isoproterenol (ISO) at a dosage of 2.0mg/kg. As expected, ISO (5min after injection) markedly increased LV contractility along with narrowed internal dimensions in all the 5 groups. The increases in LVIDd and LVIDs were similar among the groups that received LAD ligation (Figure 4 and Table 2). Additionally, the changes in the rates of LVIDd EF and FS in AD-miR-145 group were significantly lower than those in HF and AD-EGFP groups, which indicated that miR-145 could induce a reduction in β-adrenergic stimulation. One thing we must mention is that, the relatively lower change rate in sham group does not necessarily mean MI group was more vulnerable to βAR stimulation. The following reasons might be explanations: 1) EF at baseline in sham group maintained at a higher level (86.25±2.13%), while EF in the presence of ISO reaches its ultimate status (98.50±0.09%), the value of change rate in sham group might be computationally relatively lower; 2) It is because the high vulnerability to βAR stimulation of sham-operated rats that led their hearts beat to the top efficiency in the presence of ISO, part of them even reach at 99%, and these hypothesis were further confirmed in Part 2.8.

**Table 2:** Echocardiography parameters (n=5 for each group).

		**LVIDd, mm**	**LVIDs, mm**	**EF, %**	**FS, %**
Sham	Baseline	7.80±0.19	3.93±0.18	86.25±2.13	51.02±2.58
	ISO	7.48±0.13	1.71±0.07	98.50±0.09	78.00±0.99
	Rate of changes, %	-4.02±1.87	-56.19±2.79	14.41±2.76	53.89±6.29
HF	Baseline	9.34±0.36^*^	7.00±0.39^*^	55.17±2.16^*^	25.29±1.35^*^
	ISO	8.05±0.26^*^	4.20±0.0.40^*^	85.67±2.92^*^	50.96±3.59^*^
	Rate of changes, %	-13.63±2.16^*^	-42.81±2.73^*^	55.53±3.92^*^	101.79±9.48^*^
AD-EGFP	Baseline	9.60±0.26^*^	7.30±0.22^*^	54.90±0.84^*^	24.24±0.44^*^
	ISO	8.41±0.22^*^	4.04±0.25^*^	86.52±1.45^*^	52.62±1.80^*^
	Rate of changes, %	-11.99±2.15^*^	-45.41±2.58^*^	58.01±3.46^*^	108.36±7.43^*^
AD-miR-145	Baseline	8.16±0.30^*#&^	5.62±0.23^*#&^	60.60±1.83^*#&^	30.20±1.39^*#&^
	ISO	7.61±0.28^#&^	3.15±0.0.35^*#&^	88.73±0.86^*^	54.07±1.08^*^
	Rate of changes, %	-5.45±1.54^#^	-44.26±3.78^*^	42.16±4.31^*#&^	80.25±7.47^*#&^
M+C	Baseline	8.54±0.14^*#^	5.99±0.16^*#^	62.17±2.34^*#^	29.83±1.56^*#^
	ISO	7.58±0.0.12^#^	3.27±0.32^*#^	89.44±1.21^*^	56.17±2.02^*^
	Rate of changes, %	-11.38±1.78^*^	-45.67±3.61^*^	44.65±4.32^*#^	89.42±5.98^*#^

^*^P<0.05, compared with sham group; ^#^P<0.05, compared with HF group; ^&^P<0.05, compared with AD-EGFP group. Data are presented as mean ± SD.

### The role of miR-145 in alteration of β-adrenergic signaling pathway

**Figure 5:**
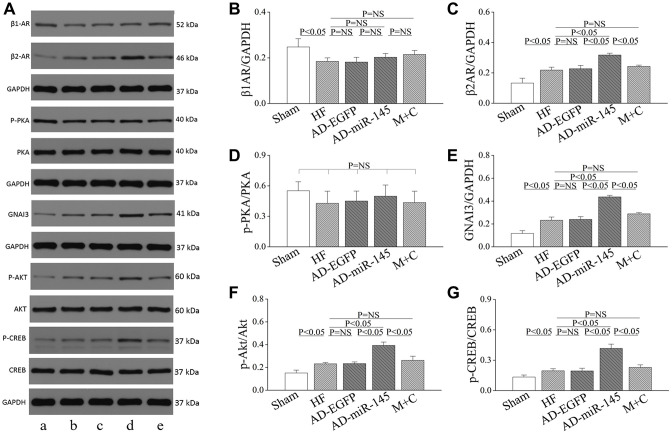
**Role of miR-145 in alteration of β-adrenergic signaling.** (**A**) Representative western blots. (**B**–**G**) quantitative analysis of the immunoreactive band displayed by bar graph (n=3). Data are presented as mean ± SD.

To clarify the potential mechanism of miR-145 mediated reduction in cardiomyocyte response to β-adrenergic stimulation, proteins involved in β-AR signaling pathway were analyzed. As illuminated in Figure 5A–5E, the level of β1AR was notably reduced while the levels of β2AR and inhibitory G protein GNAI3 were significantly increased in AD-miR-145 group. compared with those in HF and AD-EGFP groups. The redistribution of β-AR variants and elevation of GNAI3 might account for the heterogeneous response to β-adrenergic stimulation since the activity of β1/2AR-G_s_-mediated PKA cascades could be effectively restricted by Gi-based β2AR signaling [18–19]. Subsequently, in-depth investigation was conducted to uncover the modulatory role of miR-145 in G_i_-based β2AR signaling. The cAMP response element binding protein (CREB), which binds to cAMP response element in the promoter of β2AR gene, can facilitate the expression of β2AR [20]. In addition, CREB was also demonstrated to be a downstream target of protein kinase B (Akt), and a previous study indicated that miR-145 could remarkably promote the phosphorylation of Akt [21–22]. Hence, we speculated that miR-145 might promote the expression of β2AR via Akt-CREB cascades. Results of Western blotting showed that, the expression of p-Akt and p-CREB were significantly increased in AD-miR-145 group compared with those in HF and AD-EGFP groups (Figure 5A, 5F and 5G). The present data verified our hypothesis and revealed the role of miR-145 in alteration of β-adrenergic signaling pathway.

### Analysis of L-type Ca^2+^ current

### Altered I_Ca_ density, voltage dependence of I_Ca_ activation, inactivation and I_Ca_ recovery time constants

**Figure 6:**
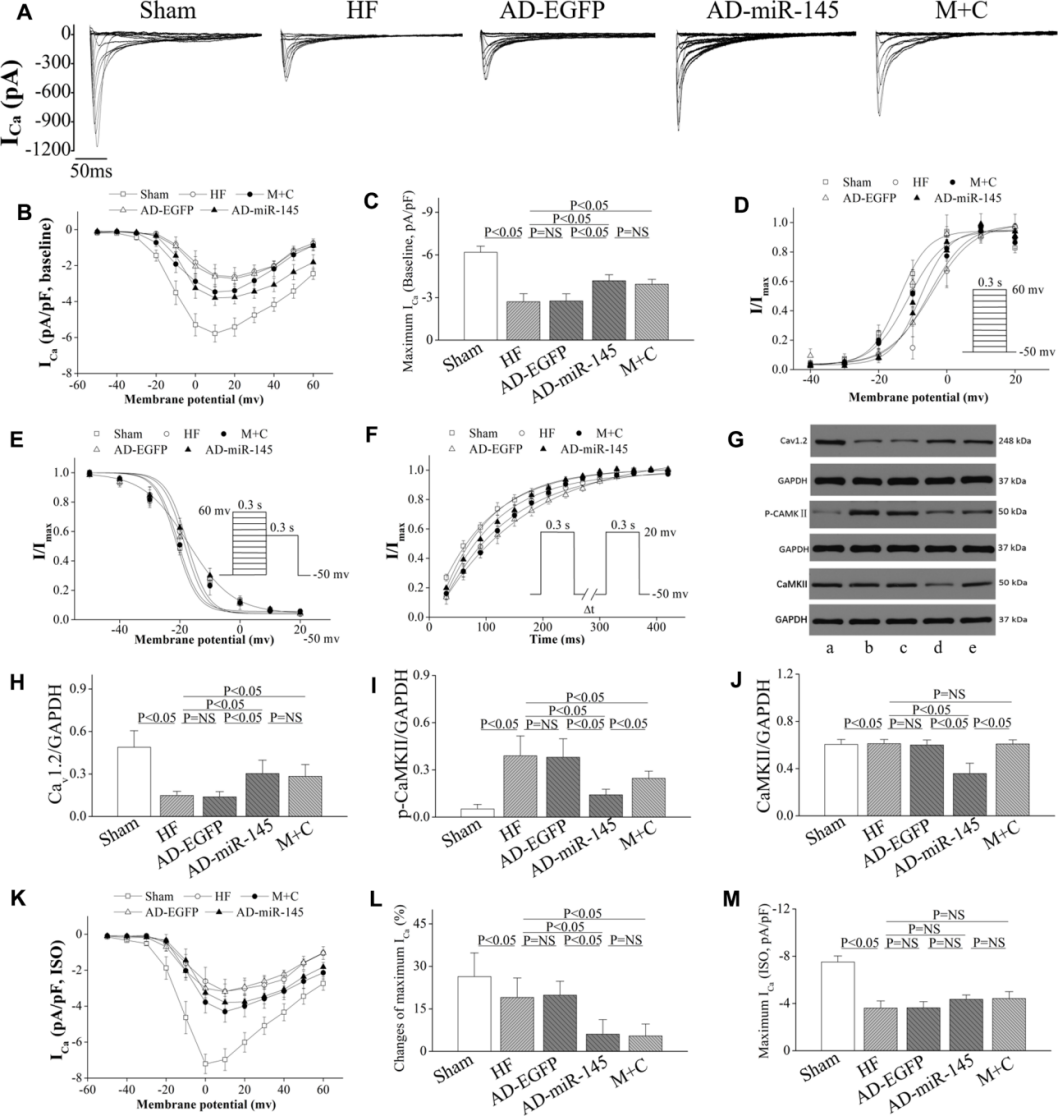
**miR-145 increased I_Ca_ density and reduced the sensitivity of HF to β-adrenergic stimulation.** (**A**) representative I_Ca_ traces recording; (**B**) I_Ca_-voltage relationship of cardiomyocytes (n≥7); (**C**) summary of peak I_Ca_ (n≥7); (**D**) the steady-state I_Ca_ activation curves (n≥7); (**E**) the steady-state I_Ca_ inactivation curves (n≥7); (**F**) the recovery curves following inactivation (n≥7); (**G**) representative western blots of Ca_v_1.2, p-CaMKII and CaMKII; (**H**–**J**) quantitative analysis of the immunoreactive band displayed by bar graph (n=3); (**K**) I_Ca_-voltage relationship in presence of ISO for each group (n≥7); (**L**) summary of peak I_Ca_ in the presence of ISO (n≥7); (**M**) changes of peak I_Ca_ for each group (n≥7); (**A**) sham group; (**B**) HF group; (**C**) AD-EGFP; (**D**) AD-miR-145 group; (**E**) M+C group. Data are presented as mean ± SD for **C**, **H**, **I**, **J**, **L** and **M**. While for **B**, **D**, **E**, **F** and **K**, data are presented as mean ± SEM.

The myocytes isolated from infarct border zone (IBZ) were used to determine the transmural variation in I_Ca_ density. In AD-EGFP and AD-miR-145 groups, cardiomyocytes stained with EGFP were selected for analysis. We found that the peak I_Ca_ (I_Ca-peak_) density was notably decreased in HF and AD-EGFP groups compared with that in Sham group (Figure 6A–6C). Conversely, AD-miR-145 and M+C treatment compensatorily enhanced I_Ca-peak_. The above-mentioned findings suggested that miR-145 had the effects of promoting I_Ca_ density. Subsequently Western blotting was carried out to investigate the molecular mechanism of miR-145-mediated increases in I_Ca_. The results of the present study showed that HF significantly activated CaMKII and promoted Cav1.2 expression (Figure 6G–6J), and this reduction in Cav1.2 expression was consistent with the diminished I_Ca_ density in HF. We have previously demonstrated that CaMKII could notably facilitate nuclear translocation of NF-κB p65 thereby resulting in reduction of Cav1.2 channel expression [23–24]. Accordingly, up-regulation of miR-145 markedly decreased the expression and phosphorylation of CaMKII thereby reversing NF-κB p65 nuclear translocation [23–24]. Collectively, the above-mentioned findings may well explain the increases in I_Ca_ observed in AD-miR-145 groups. In contrast to our results, Zhang et al [25] reported that the increased I_Ca_ in AC3-I MI mice were associated with increased PKA activity. Even so, neither total PKA expression nor its phosphorylation form has statistically changed in our experiment (Figure 5A and 5D). The inconsistent results might be attributed to the difference in CaMKII inhibition. An up-regulated miR-145 level was more likely to maintain CaMKII at a low expression level, and the remaining CaMKII might be expected to boost CaMKII activity that was sufficient to maintain its physiologic regulator role without over-activated or inhibited. This largely but incompletely suppression of CaMKII activity did not cause a compensatory increase in PKA activity (Figure 5A and 5D).

The activation conductance variable (I/I_max_) was fitted by Boltzmann function [26] to determine the activation kinetics. As displayed in Figure 6D, activation curves were significantly right-shifted in HF and AD-EGFP groups. Although up-regulated miR-145 and M+C pretreatment partially reversed HF-induced right-shifted activation curve, no significant difference was observed on activation kinetics among the groups that received LAD ligation (Table 3). Afterward, a double-pulse protocol was implemented to determine I/I_max_ for the voltage-dependent inactivation of I_Ca_. This variable was further fitted to the Boltzmann function to indicate inactivation kinetics. No significant difference was found in groups that received LAD ligation (Table 3), while the mean values of fast and slow time constants (τ1 and τ2), achieved by double exponential function [23], were robust prolonged after HF (Table 3). Eventually, the time course of I_Ca_ recovery from inactivation was fitted to mono-exponential function. As shown in Figure 6F and Table 3, the recovery time constants in AD-miR-145 and M+C groups were notably accelerated compared with those in HF and AD-EGFP groups. The miR-145-mediated acceleration of I_Ca_ recovery might also play a substantial role in the restoration of APD and electrophysiological instability.

**Table 3:** I_Ca_ characteristics (n≥7).

	**Sham**	**HF**	**AD-EGFP**	**AD-miR-145**	**M+C**
I_Ca-peak_ decay time constant, ms					
τ_1_	6.54±1.16	10.57±0.95^*^	10.46±1.13^*^	9.59±0.83^*^	10.46±0.89^*^
τ_2_	44.66±6.29	65.67±6.73^*^	65.96±9.44^*^	59.52±3.28^*^	62.01±8.51^*^
I_Ca_ activation kinetics					
V_1/2_, mV	-9.35 ±1.93	-4.18±2.26^*^	-4.84±2.65^*^	-6.67±1.54^*^	-7.17±2.78^*^
K	3.65±0.38	4.65±0.36^*^	4.79±0.38^*^	4.18±0.24^*^	4.09±0.32^*^
I_Ca_ inactivation kinetics					
V_1/2_, mV	-19.56±1.35	-18.07±1.30	-17.57±2.48	-17.32±1.25	-17.88±2.03
K	-1.45±2.01	0.16±3.06	-0.94±2.67	3.61±1.59	-0.84±2.39
I_Ca_ recovery time constant					
τ, ms	101.55	144.36	154.39	111.31	120.86
	±9.08	±7.88^*^	±13.30^*^	±11.56^#&^	±3.37^*#^

V_1/2_: half-maximal activation or inactivation; K: slope factor; τ: time constant; ^*^P<0.05, compared with sham group; ^#^P<0.05, compared with HF group; ^&^P<0.05, compared with AD-EGFP group. Data are presented as mean ± SEM.

### Overexpression of miR-145 reduced I_Ca_ vulnerability to β-adrenergic stimulation

After basal I_Ca_ was determined and reached a steady state, the given cardiomyocytes were exposed to 1μmol/l ISO to evaluate the I_Ca_ vulnerability to β-adrenergic stimulation. The results uncovered that the increase in I_Ca-peak_ induced by ISO was less prominent in AD-miR-145 and M+C groups than that of HF and AD-EGFP groups (Figure 6K–6M). It is noteworthy that miR-145-evoked G_i_-biased β2AR signaling might be related to reduction of β1AR-G_s_-PKA cascades-mediated facilitation of I_Ca_ (Figure 5A, 5C and 5D). These data demonstrated that up-regulation of miR-145 reduced the vulnerability to β-adrenergic stimulation of I_Ca_ a mouse model of HF.

### Intracellular Ca^2+^ handling in response to HF

### Altered F_0_ in cardiomyocytes

**Figure 7:**
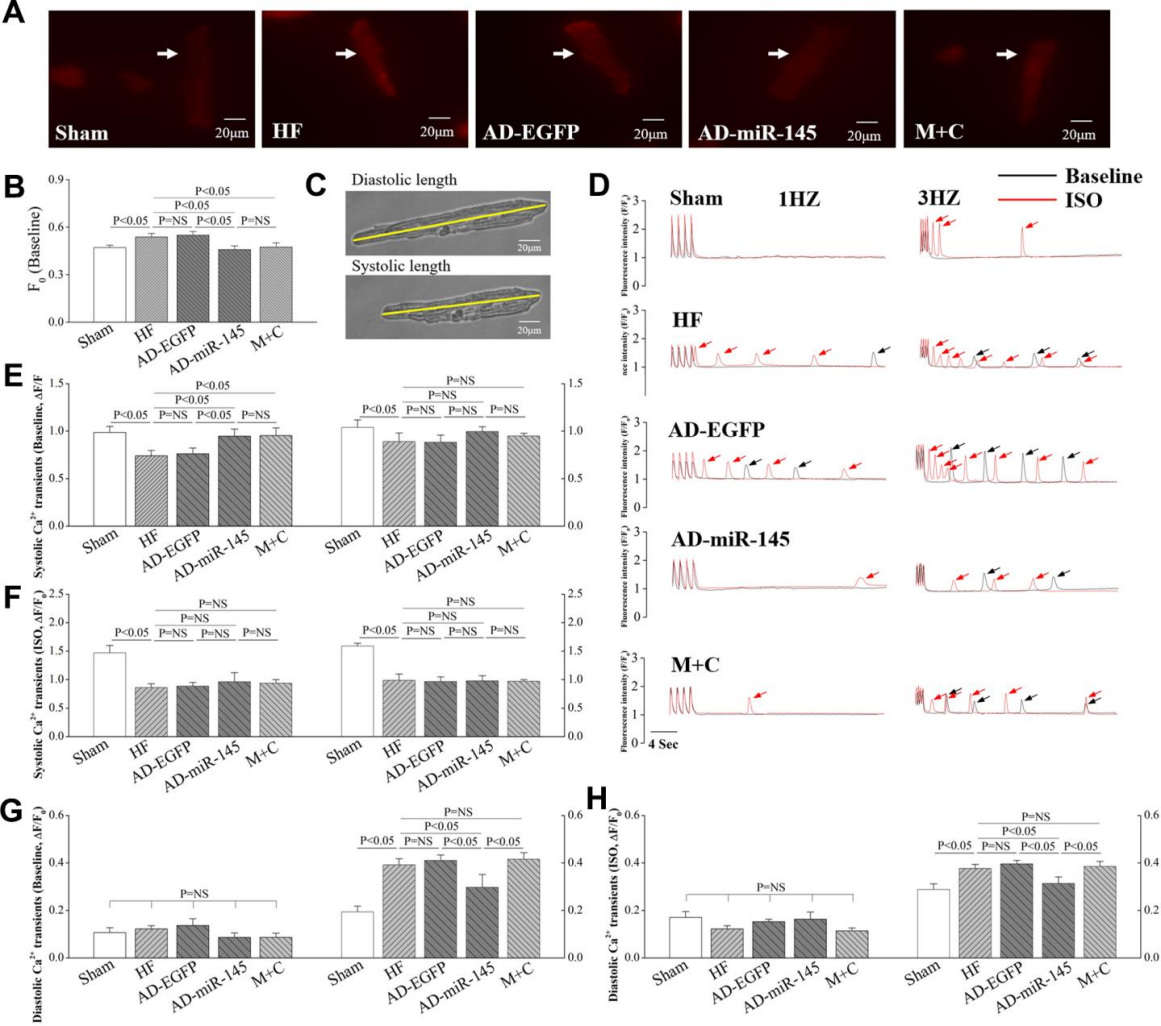
**miR-145 ameliorated HF-induced defective in Ca^2+^ homeostasis.** (**A**) representative imagines of resting fluorescence at baseline (The arrow marks cardiomyocyte stained with Rhod-2AM); (**B**) statistical analysis of F_0_ (n≥9); (**C**) representative imagines of cardiomyocytes sarcomere shortening from isolated HF hearts; (**D**) representative recording (F/F_0_) of steady-state Ca^2+^ transients followed by Ca^2+^ waves (The arrow marks Ca^2+^ wave); (**E**–**H**) statistical analysis of Ca^2+^ transients in systole and diastole (Baseline and in presence of ISO, n≥9); (**I**–**J**) statistical analysis of cell shortening (Baseline and in presence of ISO, n≥9). Data are presented as mean ± SD.

F_0_ represents intracellular cytosolic Ca^2+^ concentration in resting-state. In AD-EGFP and AD-miR-145 groups, cardiomyocytes stained with EGFP were chosen for analysis of Ca^2+^ homeostasis. As illustrated in Figure 7A and 7B, F_0_ in HF and AD-EGFP groups exhibited a higher level compared with that in Sham group. However, F_0_ was significantly diminished in AD-miR-145 and M+C groups. The above-mentioned finding indicated that AD-miR-145 and M+C treatment could effectively decrease Ca^2+^ leakage of cardiomyocytes in resting state.

### The frequency of Ca^2+^ transient

We simultaneously recorded Ca^2+^ transients and cardiomyocytes shortening in response to different stimulation rates at 1~3 Hz. The results showed that systole Ca^2+^ transients were markedly decreased in HF and AD-EGFP groups at 1Hz (Figure 7D and 7E). However, systole Ca^2+^ transients were remarkably higher in miR-145 and M+C groups than those in HF group at 1Hz. It is well-known that Ca^2+^-induced Ca^2+^ release plays a crucial role in cardiac contraction. When the release of intracellular Ca^2+^ is triggered by I_Ca_, the myofilament is driven to contracted [27], and this phenomenon is also known as excitation-contraction coupling. Therefore, the decreased Ca^2+^ transients in the current study were at least partly related to the corresponding reduction in I_Ca_ (Figure 6A and 6B), which might promote Ca^2+^ released from SR. However, such advantage of systole Ca^2+^ transients in AD-miR-145 and M+C groups at 1Hz were not observed at 3Hz. We speculated that frequency-dependent activation of CaMKII might be an explanation, activated CaMKII could further modulate PLB (Thr17)/SERCA2a cascades so as to promote sarcoplasmic reticulum (SR)-Ca^2+^-re-uptake and then increase systole Ca^2+^ transients. In addition, systole Ca^2+^ transients were dramatically increased in all the groups in the presence of ISO (Figure 7F). It also was noted that β-adrenergic stimulation induced an increase in I_Ca_, which could promote SR Ca^2+^ release, might account for these changes. Moreover, β-adrenergic stimulation could further activate CaMKII and PKA thereby facilitating SR-Ca^2+^-re-uptake as well as promoting systole Ca^2+^ transient.

**Figure 8:**
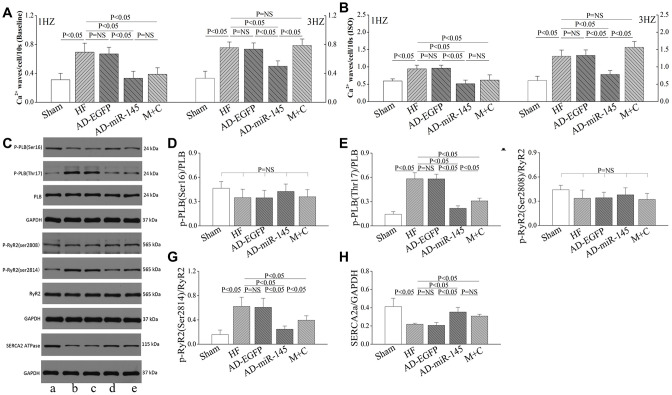
**miR-145 ameliorated HF-induced defective in Ca^2+^ homeostasis.** (**A**–**B**) statistical analysis of Ca^2+^ waves (Baseline and in presence of ISO, n≥9); (**C**) Representative western blots; (**D**–**H**) quantitative analysis of the immunoreactive band displayed by bar graph (n=3). (a) sham group; (**B**) HF group; (**C**) AD-EGFP; (**D**) AD-miR-145 group; (**E**) M+C group. Data are presented as mean ± SD.

We further analyzed diastole Ca^2+^ transients with the same filed stimulation protocol. With the increase of stimulation frequency, diastolic Ca^2+^ transients in HF and AD-EGFP groups were markedly increased at 3Hz compared with those in Sham group (Figure 7D, 7G). However, HF-induced diastole Ca^2+^ transients augment were significantly attenuated by up-regulation of miR-145 at 3Hz. The above-mentioned results indicated that a superior extent of frequency-dependent acceleration of relaxation (FDAR) could be observed after miR-145 treatment. We considered that miR-145-mediated depression on CaMKII over-activation might be an explanation. Especially when CaMKII was highly activated after HF, such changes could finally aggravate the phosphorylation of RyR2, Ca^2+^ leak and deterioration in FDAR. Similar results were observed when given cardiomyocytes were exposed to ISO (Figure 7H). ISO could notably facilitate β1AR-G_s_-PKA signaling thereby leading to hyperactivation of RyR2 at Ser2808 and deterioration in Ca^2+^ leak. However, such disadvantages could be influenced by miR-145 treatment, since miR-145-evoked β2AR-G_i_ signaling could restricts the activation of β1AR-G_s_-PKA cascades. Previous studies demonstrated that the phosphorylation of CaMKII is necessary for FDAR, and suppression of CaMKII activity may result in diastolic dysfunction [28]. Our findings may be justified as follows:: 1) The remaining activity of CaMKII might be sufficient to accommodate the needs of FDAR in AD-miR-145 group; 2) Diastolic Ca^2+^-elimination due to SR-Ca^2+^-re-uptake via PLB/SERCA2a cascades played a role in facilitating FDAR. As shown in Figure 8C and 8H, the expression of SERCA was significantly higher in AD-miR-145 group than that in other groups that received LAD ligation, suggesting that cardiomyocytes in AD-miR-145 group outperformed those in diastolic Ca2+-elimination.

Consistent with the changes of Ca^2+^ transients, FS were significantly reduced at different frequencies of pacing (Figure 7I and 7J) in HF and AD-EGFP groups, which indicated that HF could dramatically impair cardiac reserve. However, FS in M+C group was significantly higher at 1Hz compared with HF group, while such an advantage was not noted at 3Hz due to an undesirable augment in diastolic Ca^2+^ transients. However, up-regulation of miR-145 noticeably improved FS at a frequency of 3Hz compared with that in M+C group, and at all stimulation frequencies compared with HF and AD-EGFP groups. These outcomes demonstrated that over-expression of miR-145 could ameliorate HF-impaired cardiac reserve and a superior cardiac performance was achieved at a higher frequency of pacing or workload conditions compared with traditional M+C treatment.

### The incidence of Ca^2+^ waves

Ca^2+^ waves could directly reflect the degree of Ca^2+^ sparks and Ca^2+^ leak, and were calculated following 30s after subjecting to different frequencies of pacing. We found that HF significantly promote the frequency of Ca^2+^ waves at 1Hz (Figure 8A), and with the increase of pacing frequency, Ca^2+^ waves were remarkably increased in HF, AD-EGFP and M+C groups compared with AD-miR-145 group at 3Hz (Figure 8A). The frequency-dependent activation of CaMKII may account for the promotion of Ca^2+^ waves, in which CaMKII-mediated phosphorylation of RyR2 may lead to an increase in SR Ca^2+^ leak. The western bolt further confirmed our results. As shown in Figure 8C, 8F and 8G, expression of phosphorylated RyR2 at Ser2814 were significantly increased in HF and AD-EGFP groups while up-regulation of miR-145 and M+C treatment markedly suppressed the activation of RyR2 (Ser2814).

Therefore, it can be concluded that up-regulation of miR-145 is capable of restraining Ca^2+^ waves or even Ca^2+^ sparks and Ca^2+^ leak, thereby ameliorating cardiac performance and arrhythmia risk. Furthermore, these cardioprotective effects might be somewhat superior to M+C treatment. The experiment was repeated in the presence of ISO and results were similar to those of Ca^2+^ waves that were observed at baseline (Figure 8B), which indicated that cardiomyocytes treated with AD-miR-145 were more tolerant to high frequency field stimulation as well as β-adrenergic stimulation.

## DISCUSSION

The results of the present study revealed cardioprotective role of miR-145 in HF. It was unveiled that up-regulation of miR-145 could notably promote β2AR-GNAI3 signaling and suppress HF-induced over-activation of CaMKII cascades thereby preventing adverse cardiac remodeling response including myocardial fibrosis, cardiac dysfunction and malignant arrhythmia. The results indicated that miR-145 could be a biological specific target to effectively modulate the activity of CaMKII, and might have potentially broad implications for treatment of cardiac anti-remodeling after HF.

### Alteration in β-adrenergic signaling pathway

Numerous evidences have revealed the pivotal roles of β1AR and β2AR in determining the fate of cardiomyocytes [8, 29]. A number of scholars highlighted the cardioprotective effects of β2AR-G_i_ coupling on inhibition of maladaptive remodeling induced by β-adrenergic stimulation [30]. However, β2AR-G_i_ coupling may functionally restrict β1/2AR-G_s_-mediated cAMP/PKA cascades thereby leading to dysfunction of cardiac performance and βAR-mediated inotropic response in HF [19]. The results of the current research showed that over-expression of miR-145 significantly promote the expression of β2AR and GNAI3 via activation of Akt-CREB signaling, and cardiac response to βAR stimuli (induced by ISO) was diminished. It is well-known that attenuated response to βAR activation is associated with a reduction in cardiac reserve [23]. However, cardiac performance, I_Ca_ amplitude and Ca^2+^ transients in AD-miR-145 group remained superior or not inferior to that of HF and AD-EGFP groups at baseline or in the presence of ISO. On the contrary, electrophysiological instability and Ca^2+^ mishandling brought by βAR stimulation was notably attenuated in AD-miR-145 groups compared with those in HF and AD-EGFP groups. It can be therefore concluded that, miR-145 transfection created an appropriate balance between β1AR-G_s_ and β2AR-G_i_ signaling as well as CaMKII cascades in HF.

### miR-145 attenuated cardiac structural remodeling

The findings of the present study also showed that miR-145 was notably decreased with pronounced activation of CaMKII 4 weeks after HF. Stimulation of β2AR-GNAI3 coupling has been proven to play a cardioprotective role in HF, which was manifested by depression on loss of cardiomyocytes, cardiac apoptosis and maladaptive remodeling [30]. Results of previous studies confirmed our data that miR-145 treatment facilitated β2AR-GNAI3 signaling and dramatically decreased infarcted size. A prior study demonstrated that chronic CaMKII inhibition plays a protective role against HF-induced cardiac structural remodeling by attenuating pro-inflammatory chemoattractant signaling [31]. In addition, transgenic over-expression of CaMKIIδ was observed to alter intracellular Ca^2+^ homeostasis, which led to cardiac remodeling in mice in vivo [32], while deletion of CaMKIIδ relieved the progression of HF. Recently, CaMKII was proven to be a downstream target of miR-145, and up-regulated miR-145 could effectively suppress pathological of over-activation CaMKII [12, 33]. Consistent with our results, up-regulation of miR-145 intensively inhibited the expression and phosphorylation of CaMKII, and subsequently alleviated HF-triggered LV remodeling via diminishing myocardial fibrosis and infarct size as well as promoting cardiac contraction. Furthermore, miR-145 was also reported to exert additional infarct size-reducing effects by accelerating autophagy [21] and down regulating PDCD4 [34].

### miR-145 suppressed HF-induced electrophysiological instability

APD dispersion is a representative factor leading to malignant arrhythmia. The findings of the current research unveiled that APD_90_ was dramatically shortened at IBZ after MI, which indicated an increased susceptibility to VT [35–36]. These results were further confirmed by PES, as evidenced by elevated arrhythmia scores. However, miR-145 over-expressed hearts markedly reversed HF-induced electrophysiological instability. APD is closely associated with cardiac repolarization which is mainly determined by the balance of inward and outward membrane current [37]. HF strikingly impairs L-type Ca^2+^ channel functions and thereby a relatively higher outward K^+^ current can finally promote cardiac repolarization, thereby resulting in shortened APD. We therefore assessed the effect of miR-145 on I_Ca_. As expected, miR-145 significantly increased I_Ca_ density which might partly contribute to the prolongation of APD after HF. Previous studies pointed that CaMKII could promote nuclear translocation of NF-κB p65, during which process the latter inhibited the expression of Ca_v_1.2 and finally led to decrease in I_Ca_ [23, 38]. However, miR-145 can reverse the down-regulation of Ca_v_1.2 due to excessive activation of CaMKII and decreased APD dispersion thereby protecting against maladaptive electrical remodeling after HF. However, miR-145-evoked β2AR-G_i_ coupling might depress β1AR-G_s_-PKA cascades, exerting a downward influence on I_Ca_, and no significant differences were observed in the expression of PKA or phosphorylated PKA among the groups that received LAD ligation.

### miR-145 improved intracellular Ca^2+^ homeostasis

Intracellular Ca^2+^ homeostasis is widely accepted as a major factor that may lead to cardiac dysfunction and malignant arrhythmia. HF-induced chronic over-activation of CaMKII noticeably promotes the phosphorylation of RyR2, SR Ca^2+^ leak and intracellular Ca^2+^ overload. In the meantime, SERCA2a-dependent Ca^2+^ re-uptake, Ca^2+^ transients, and SR-Ca^2+^ content, and cell shortening are impaired, and these changes contributed to the reduction threshold of APD alternans [39] and may constituted a the pathophysiological basis for HF and arrhythmia [18, 40]. The present study supported these findings via an impaired electrophysiological stability, in addition to cardiac systolic and diastolic functions after HF. In addition, such disadvantages were particularly obviously at 3HZ. Hyperphosphorylation of RyR2 due to frequency-dependent activation of CaMKII further aggravate the disability of cardiac diastole and SR-Ca^2+^ leak. In addition, inappropriate elevation of SR Ca^2+^ leak can finally promote Ca^2+^ waves and arrhythmogenic inward Na^+^/Ca^2+^ exchange (NCX1) current, leading to delayed afterdepolarization and VT. Consistent with finding of the present study, Lu et al. [41] demonstrated that CaMKII could further promote NCX1 expression while attenuate the level of SERCA, thereby disturbing intracellular Ca^2+^ homeostasis in HF models in vivo. However, up-regulation of miR-145 relieved the over-activated CaMKII-mediated defection of intracellular Ca^2+^ homeostasis, indicating that miR-145a the potential to ameliorate Ca^2+^ mishandling after HF. More importantly, this largely incomplete inhibition could partly preserve the activity of CaMKII, which was required to maintain its physiological functions, and we it may be a more clinically relevant approach that can be applied in HF treatment.

Of note, traditional anti-remodeling treatment (combination of ACEIs and β-AR antagonist) remarkedly reduced HF-induced phosphorylation of CaMKII, thereby attenuating intracellular Ca^2+^ oscillation. However, frequency-dependent activation of CaMKII could not be effectively inhibited by M+C treatment. These phenomena prompted us the fact that in certain unusual circumstances such as workload and tachycardia. Hence, it can be concluded that M+C treatment may not achieve expected improvement on cardiac performance and arrhythmia risk owing to frequency-dependent hyperactivation of CaMKII. On the contrary, the disadvantages of M+C treatment may be elucidated by upregulation of miR-145 as evidenced in the current study.

### The role of miR-145 in HF treatment

1) Traditional medicine (combination of ACEIs and βAR antagonist) could improve the prognosis of HF by suppressing cardiac remodeling. However, the performance of cardiomyocytes in M+C group was not highly appropriate as evidenced by a deterioration in diastolic dysfunction and incidence of Ca^2+^ waves at a high pacing rate. This phenomenon might be attributed to the differential inhibition of CaMKII, and the positive role of miR-145 might be related to a better prognosis for HF patients, especially in response to workload and stress conditions; 2) The clinical application of combination of ACEIs and β-AR antagonist might be limited by various contraindications, such as bradycardia, renal insufficiency and hypotension; 3) miR-145 might provide additional anti-atherosclerosis effects, which was manifested by limiting atherosclerotic plaque morphology, cellular composition and shifting from rupture plaque to stable one [42–43].

### Limitations

Cardiac K^+^ currents play a substantial role in determination of APD and hyperactivation of CaMKII in order to impair the function of K^+^ channel, thereby causing dispersion of APD [44]. Analysis of K^+^ currents with corresponding channel protein will be more effective to figure out the anti-electrical remodeling effects of miR-145.

## CONCLUSION

**Figure 9:**
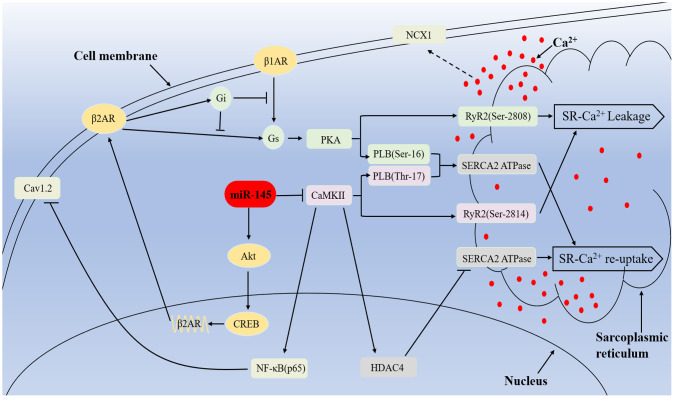
**Mechanisms of miR-145-mediated cardioprotective effects against MI.** miR-145 promotes the expression of β2AR via activation of Akt/CREB cascades and enhances β2AR-G_i_ activity, which in turn restricts β1/2AR-G_s_ signaling and leads to reduced response to β-adrenergic stimuli. Furthermore, MI-induced hyperactivation of CaMKII promote RyR2-mediated Ca^2+^ release and result in activation of NCX1, which finally leads to delayed depolarization. At the meantime, CaMKII suppressed SERCA2a and Ca_v_1.2 expression via HDAC4 and NF-κB (p65) pathway respectively, thus impair Ca^2+^ re-uptake, excitation-contraction coupling and cardiac performance. However, miR-145-mediated inhibition on CaMKII expression partially reversed the related disadvantages.

In summary, the present study demonstrated for the first time that miR-145 could prevented cardiac structure and electrical remodeling via ameliorating myocytes fibrosis, cardiac contractility, electrophysiological instability and intracellular Ca^2+^ homeostasis in response to HF. These cardioprotective effects may be predominantly due to the alteration of βAR signaling and chronical inhibition of hyperactivated CaMKII cascades (Figure 9). Considering the unique characteristics of miR-145 under workload and stress conditions, it may serve as a new therapeutic target for the treatment of HF in the future.

## MATERIALS AND METHODS

### Animals

Male Sprague-Dawley rats (aged, 7-8 weeks; weight, 230-260g) were acclimatized to laboratory conditions on a 12/12h light/dark cycle with ad libitum access to food and water. The experimental animal protocols were carried out in accordance with the Guide for the Care and Use of Laboratory Animals (NIH, Bethesda, MD, USA), and were approved by the Institutional Animal Care and Use Committee of Wuhan University.

### HF model and experimental design

HF models were established as previously described [45–46]. Briefly, thoracotomy and pericardiotomy were conducted under anesthesia to exposure heart. Subsequently, a 6-0 silk suture was snugly secured approximately 2mm below the left atrial appendage to ligate the left anterior descending coronary artery (LAD). A remarkable elevation of ST segment and pillarization of epicardium was considered as successful modeling of myocardial infarction (MI).

Rats were randomly assigned into five groups: A. Sham group, the sham-operated control group; B. HF group, intragastric administration of normal saline for 4 weeks after ligation of LAD; C. AD-EGFP group, transfection with AD-EGFP after ligation of LAD; D. AD-miR-145 group, transfection with AD-miR-145 after ligation of LAD; E. M+C group, intragastric administration of metoprolol (Sigma-Aldrich, St. Louis, MO, USA; 250mg/kg/d, dissolved in normal saline) and captopril (Sigma-Aldrich, St. Louis, MO, USA; 100mg/kg/d, dissolved in normal saline) for 4 weeks after ligation of LAD. Rats were sacrificed 4 weeks after MI, and BW, LW and HW were calculated.

### Transfection of AD-miR-145 into the heart

Adenovirus carries an enhanced green fluorescent (EGFP) gene. The heart was exposed as described above. Besides, 100μl of AD-miR-145 (1×10^11^PFU, GeneChem Co.,Ltd, Shanghai, China) or AD-EGFP(1×10^11^PFU, GeneChem Co.,Ltd, Shanghai, China) were immediately injected into 5 sites of IBZ using a 33-gauge needle after LAD ligation. Rats were allowed to recovery after injection.

### PCR (RT-qPCR)

Total RNA was isolated using TRIzol Reagent (Life Technologies, Carlsbad, CA, USA) according to the manufacture’s protocol. RT-qPCR was performed on an ABI-PRISM 7900 Sequence Detection System (ELK Biotechnology, China). The primers used in this study were as follows for: miR-145-5p (forward): TGTCCAGTTTTCCCAGGAATC; miR-145-5p (reverse): CTCAACTGGTGTCGTGGAGTC; U6 (forward): CCT GCTTCGGCAGCACAT, U6 (Reverse): AACGCTTC ACGAATTTGCGT. The relative expression of miR-145 was normalized to U6 and calculated using the 2^-ΔΔCt^ method.

### Echocardiography

Echocardiography was conducted on lightly anesthesia rats 4 weeks after HF as previously described [23, 47]. Echocardiography parameters were measured at the midpapillary level from well-aligned M-mode images to obtain variables as follows: LVIDd, LVIDs, LVEF and FS. The protocol was repeated 5min after intraperitoneal injection of ISO (2mg/kg).

### Histological evaluation

Heart samples were paraffin-embedded 4 weeks after HF, then the hearts were sectioned laterally into several slices (5μm) at left ventricle papillary muscle level and were stained with hematoxylin and eosin (H&E) or Masson’s trichrome. Finally, the stained sections were visualized at 4× and 400×magnification for morphological analysis. For CVF, 5 random fields per slice from IBZ were analyzed. Infarct size (fraction of the infarcted LV, IS) was quantified as the average of all slices and expressed as percentage of length [48].

### ECG

Surface lead II ECG was recorded before programmed electrical stimulation (PES) on lightly anesthesia rats 4 weeks after HF. ECG signals were recorded at 1 kHz sampling rate continuously, and a stable periods of 5 min were measured by LabChart 7.8 (ADInstruments, Sydney, Australia) to obtain RR interval, PR interval, QRS duration and QT interval. The corrected QT (QTc) interval was calculated using Bazett’s formula [26]: QTc= QT/(RR/100)^1/2^.

### PES

Rat was lightly anesthetized, and the heart was exposed as described above. ECG (LabChart 7.8, AD instruments) was continuously monitored during PES procedure and the protocol for PES was carried out according to published methods [49]. Briefly, a custom-made platinum electrical stimulator was placed at the IBZ. A drivetrain of 8 stimuli of S1 at a pacing cycle length (PCL) of 120ms was delivered, followed by 1-3 extrastimuli (S2, S3 and S4) at 2ms decrements until effective refractory period was reached. To quantify the inducibility of ventricular tachyarrhythmias (VT), an arrhythmia scoring system was implemented as follows [50]: 0, noninducible preparations; 1, nonsustained VT induced with three extrastimuli; 2, sustained VT induced with three extrastimuli; 3, nonsustained VT induced with two extrastimuli; 4, sustained VT induced with two extrastimuli; 5, nonsustained VT induced with one extrastimuli; 6, sustained VT induced with one extrastimuli; 7, VT induced during the eight paced beats; 8, cardiac arrest without pacing. VT lasted > 15beats or lasted around 6-15 beats was defined as sustained VT or nonsustained VT respectively. The highest score was calculated when multiple arrhythmias were induced.

### Monophasic action potential (MAP) recording

Langenbdorff-perfused heart was prepared using Tyrode’s solution (mmol/L: 135 NaCl, 5.4 KCl, 1.8 CaCl_2_, 1.0 MgCl_2_, 0.3 Na_2_HPO_4_, 10 HEPES, and 10 glucose; pH adjusted to 7.35 with NaOH) as previously described [51]. A custom-made Ag-AgCl electrode was used for MAP recording at the IBZ, and a paired platinum electrical stimulating electrode was placed at the basal surface of the right ventricle to deliver delivered 2ms square-wave stimulation pulses. APD signals were amplified, band-pass filtered and recorded between 0.3HZ and 1HZ by a dual bioamplifier (LabChart 7.8,; ADInstruments, Sydney, Australia). A drivetrain of 100 stimuli of S1 set with a PCL shorten from 150ms to 100ms at 10ms decrements and then to 50ms at 5ms decrements was delivered. APD_90_ was defined as the average of zero-phase depolarization to the 90% repolarization time for 8 successive MAPs. APD alternans was considered when alternate APD_90_ differ by 5% over at least 10 beats [52]. When multiple ADP_90_ alternans were induced, the longest PCL was calculated as threshold of ADP_90_ alternans.

### Cell isolation

Four weeks after MI, ventricular myocytes from IBZ were isolated by enzymatic digestion after perfusion of heart with type II collagenase (Worthington Biochemical Corporation, Lakewood, NJ, USA) using a previously described method [53]. Isolated myocytes were stored in Ca^2+^-free Tyrode’s solution plus 1 mg/ml bovine serum albumin at room temperature. Experiments were completed within 5h after isolation of ventricular myocytes.

### Patch-clamp recording

Whole-cell configuration of the patch-clamp technique was used for current clamp and voltage-clamp electrophysiological recordings using the EPC-9 patch-clamp amplifier (HEKA Elektronik, Lambrecht, Germany). Single cardiomyocyte was placed in an experimental chamber that was perfused with external solution (mmol/L: 100 choline chloride, 35 NaCl, 5.4 KCl, 1.8 CaCl_2_, 1.0 MgCl_2_, 0.33 NaH_2_PO_4_, 10 HEPES, 10 glucose, 0.1 BaCl_2_·2H_2_O, 5 4-aminopyridine; pH adjusted to 7.35 with NaOH) and mounted under an inverted fluorescence microscope (IX70; Olympus, Tokyo, Japan). The detection of I_Ca_ was carried out using a glass microelectrode (maintained with a 3.0-5.0MΩ resistance and perfused with pipette solution (mmol/L): 120 CsCl, 1.0 CaCl_2_, 5.0 MgCl_2_, 5.0 Na_2_ATP, 11 EGTA, 10 HEPES and 11 glucose, pH adjusted to 7.35 with CsOH) at 20-25°C. The mean capacitance and series resistance of given cardiomyocyte was maintained below 25 MΩ. The protocol was ditally sampled at 10kHZ with low-pass filtered at 1kHZ and was repeated in the presence of 1μmol/l ISO.

The following experiment was carried out for the detection of voltage dependence of I_Ca_: a holding potential of -50mV was used and steps of 300-ms duration test pulse from -50mV to +60mV in 10-mV were preceded by a 50-ms prepulse of -50mV. A double-pulse protocol (300ms conditioning pulse at different potential ranging from -50mv to +60mv, followed by a 300ms test pulse to +10mv) was applied to determine the voltage-dependent inactivation of I_Ca_. The recovery time from inactivation was evaluated using the following double-pulse protocol: two identical pulse with holding potential from -50mV to +20mV for 300ms were applied, followed by a variable time from 30 to 480ms at an interval of 30ms.

### Electrical field stimulation experiments

Isolated myocytes were incubated with Ca^2+^ indicator Rhod-2AM (5μmol/L, Invitrogen, Carlsbad, CA, USA) in Tyrode’s solution at 37°C for 50 min. The mixture was resuspended every 5min. Subsequently, the loaded cardiomyocytes were de-esterified for 10min and stored in dye-free Tyrode’s solution. Rod-shaped myocytes with clear striations, and stable contractions were selected for cell shortening, resting fluorescence (F_0_), Ca^2+^ transients and Ca^2+^ waves analyzing. The fluorescence intensity of Rhod-2AM (measured at 579 nm upon excitation at 549 nm) was acquired by whole-cell Ca^2+^ imaging with a Leica AF6000 fluorescence microscope (Leica Microsystems Inc, Germany). A custom-made chamber equipped with paired platinum stimulating electrodes was used to dissipate field stimulation. Cardiomyocytes were electrically paced at a range of 1 and 3Hz with 8 pulses followed by a 30s pause, in which the frequency of Ca^2+^ waves were quantified in that period. Fluorescence was normalized to F_0_. Cell length under contraction (systolic length) and relaxation (diastolic length) was analyzed using LAS X. FS was calculated according to the following equation [54]: (diastolic length-systolic length)/ diastolic length×100%. The experiment was repeated in the presence of 1μmol/l ISO.

### Protein analysis

Heart samples were immediately stored in -80°C before western blot analyses performed. Western blot was performed as previously described [7]. Briefly, proteins were extracted from IBZ and the concentrations of protein were acquired using a BCA protein concentration assay kit (Sigma Aldrich, Germany). Equal amounts of protein (40μg) were electrophoresed on 10% SDS-PAGE and were transferred to PVDF membranes, which were then incubated with corresponding primary antibodies overnight at 4°C. Subsequently, membrane strips were extensive washed by TBST and a secondary antibody was applied to detect the primary antibody binding. Protein bands were analyzed with a chemiluminescence method, and AlphaEaseFC software processing system (Alpha Innotech, USA) was used to calculate the optical density of target bands. The following primary antibodies were used for western blot analysis: GAPDG (1:10000, Abcam), p-PLB-Ser16 (1:1000, Thermo Fisher Scientific), p-PLB-Thr17 (1:500, Badrilla), PLB (1:1000, Badrilla), p-RyR-Ser2808 (1:200, Abcam), p-RyR-Ser2814 (1:500, Badrilla5), RyR2 (1:500, Abcam), SERCA2-ATPase (1:3000, Abcam), p-PKA (1:1000, Abcam), β1AR (1:500, Bioss), β2AR (1:500, Bioss), GNAI3 (1:2000, Abcam), p-CaMKII (1:1000, Abcam), CaMKII (1:500, Abcam), Ca_v_1.2 (1:200, Abcam), collagen I (1:1000, Abcam), collagen III (1:500, Abcam), α-SMA (1:10000, Abcam), p- protein kinase B (Akt, 1:1000, Cell Signaling Technology), Akt(1:3000, Cell Signaling Technology), p-cAMP response element binding protein (CREB, 1:500, Cell Signaling Technology), CREB (1:1000, Cell Signaling Technology).

### Statistical analysis

Statistical analysis was performed using SPSS 19.0 software (IBM, Armonk, NY, USA). Continuous variables were expressed as mean ± standard deviation (SD) or mean ± standard error (SEM). Differences between two groups were analyzed using paired and unpaired tow tailed t-test, while those difference among multiple groups were compared using one-way analysis of variance (ANOVA) followed by post hoc test. If the normality and variance requirements were not satisfied, the Mann-Whitney U test was employed. P < 0.05 was considered statistically significant.
